# A mitochondrial ROS pathway controls matrix metalloproteinase 9 levels and invasive properties in RAS‐activated cancer cells

**DOI:** 10.1111/febs.14671

**Published:** 2018-10-13

**Authors:** Kazunori Mori, Tetsu Uchida, Toshihiko Yoshie, Yuko Mizote, Fumihiro Ishikawa, Masato Katsuyama, Motoko Shibanuma

**Affiliations:** ^1^ Division of Cancer Cell Biology Department of Pharmaceutical Sciences Showa University School of Pharmacy Tokyo Japan; ^2^ Radioisotope Center Kyoto Prefectural University of Medicine Japan

**Keywords:** HIC‐5, metastasis, MMP9, NOX4, ROS

## Abstract

Matrix metalloproteinases (MMPs) are tissue‐remodeling enzymes involved in the processing of various biological molecules. MMPs also play important roles in cancer metastasis, contributing to angiogenesis, intravasation of tumor cells, and cell migration and invasion. Accordingly, unraveling the signaling pathways controlling MMP activities could shed additional light on cancer biology. Here, we report a molecular axis, comprising the molecular adaptor hydrogen peroxide‐inducible clone‐5 (HIC‐5), NADPH oxidase 4 (NOX4), and mitochondria‐associated reactive oxygen species (mtROS), that regulates MMP9 expression and may be a target to suppress cancer metastasis. We found that this axis primarily downregulates mtROS levels which stabilize MMP9 mRNA. Specifically, HIC‐5 suppressed the expression of NOX4, the source of the mtROS, thereby decreasing mtROS levels and, consequently, destabilizing MMP9 mRNA. Interestingly, among six cancer cell lines, only EJ‐1 and MDA‐MB‐231 cells exhibited upregulation of NOX4 and MMP9 expression after shRNA‐mediated HIC‐5 knockdown. In these two cell lines, activating *RAS* mutations commonly occur, suggesting that the HIC‐5–mediated suppression of NOX4 depends on RAS signaling, a hypothesis that was supported experimentally by the introduction of activated RAS into mammary epithelial cells. Notably, HIC‐5 knockdown promoted lung metastasis of MDA‐MB‐231 cancer cells in mice. The tumor growth of HIC‐5–silenced MDA‐MB‐231 cells at the primary sites was comparable to that of control cells. Consistently, the invasive properties of the cells, but not their proliferation, were enhanced by the HIC‐5 knockdown *in vitro*. We conclude that NOX4‐mediated mtROS signaling increases MMP9 mRNA stability and affects cancer invasiveness but not tumor growth.

## Graphical abstract

Matrix metalloproteinases (MMPs) are a group of tissue‐remodeling enzymes that have been linked to a variety of pathophysiological processes, including cancer metastasis. Here, Motoko Shibanuma and co‐authors provide new insights into the regulation of MMP9. They show that the molecular adaptor protein HIC‐5 suppresses the activation of NADPH oxidase 4 (NOX4), leading to downregulation of mitochondrial ROS levels, which in turn destabilizes MMP9 mRNA. They further demonstrate that this regulatory axis operates specifically in cancer cells harboring oncogenic mutations in H‐ or K‐ras, potentially unveiling a new therapeutic avenue for cancer therapy based on inhibition of MMPs.

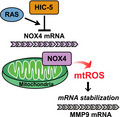

**Abbreviations**
CoQ: coenzyme Q
EGFP: enhanced green fluorescent protein
HIC‐5: hydrogen peroxide‐inducible clone‐5
hMEC: human mammary epithelial cells
hTERT: the catalytic subunit of human telomerase
Mito‐TEMPO: (2‐(2,2,6,6‐tetramethylpiperidin‐1‐oxyl‐4‐ylamino)‐2‐oxoethyl)triphenylphosphonium
Mitq: mitoquinol
MMP: matrix metalloproteinase
mtROS: mitochondria‐associated reactive oxygen species
NAC: *N*‐acetyl cysteine
NOX: NADPH oxidase
qPCR: real‐time RT‐PCR
Tet: tetracycline
Tiron: 1,2‐dihidroxybenzene‐3,5‐disulphonic acid
TPP: tripheylphosphonium

## Introduction

Matrix metalloproteinases (MMPs) are a group of zinc endopeptidases with proteolytic activity against the extracellular matrix. These have been classically recognized as tissue‐remodeling enzymes, while recent studies revealed their involvement in processing various bioactive molecules and broadened their roles 1. For cancer cells, these enzymes are indispensable to accomplish metastatic processes, such as angiogenesis, intravasation of tumor cells, migration and invasion of metastatic cells in the secondary organ, and initiation of tumor growth in the metastatic site 2. These processes are highly dependent on activity remodeling and/or degrading the extracellular matrix.

The MMP2 and MMP9 are classified into the gelatinase subgroup of the MMP family, and are thought to play important roles in metastatic processes, mainly based on their ability to degrade type IV collagen 3, 4. Increased expression of MMP9 and MMP2 has been observed in invasive and highly tumorigenic cancers, and affirmed to be associated with the relapse of disease, metastasis, and shorter overall survival 5. However, some complexity has been posed in the functions of MMPs, with antitumorigenic activities revealed in certain cases 6. This finding has alerted the medical community regarding the general use of MMP inhibitors for cancer therapy. In addition, clinical trials investigating the efficacy of early generation inhibitors have failed due to dose‐limiting toxicity or insufficient beneficial outcomes 7, 8.

Nevertheless, MMPs remain attractive therapeutic targets, considering the number of clinical areas, including cancer therapy that could benefit from MMP inhibition. Conceptually, new approaches are imperatively necessary to circumvent the above caveats and setbacks, and develop truly effective inhibitors as next‐generation therapeutic agents. Intriguingly, the expression pattern of MMPs is spatiotemporally restricted and/or extremely limited. Given the diversity of their substrates, the protease activity has to be closely controlled. Thus, MMP levels are extremely low under normal conditions and highly inducible in particular circumstances. Under such conditions, MMP expression is stimulated by relevant inducers 9. Harnessing such a specific inducing activity for the regulation of MMP expression may provide the option for more restricted interruption of MMP action than inhibition of overall enzymatic activity.

The MMP inducers include intracellular reactive oxygen species (ROS), which upregulate MMPs by regulating both gene expression and proenzyme activation 10, 11. In cancer cells, ROS were reported to mediate an increase in MMP activity by growth factor stimulation 10. In the present study, we report the specific molecular mechanism for the control of mitochondria‐associated ROS (mtROS) levels and regulation of MMP9 expression. The system comprises the molecular adaptor HIC‐5 (hydrogen peroxide‐inducible clone‐5) 12, 13 and the ROS‐generating enzyme NOX4 (NADPH oxidase 4), and is designated as the HIC‐5‐NOX4‐mtROS axis. HIC‐5 suppresses NOX4 expression at the mRNA level, leading to lower mtROS levels and accelerating the decay of MMP9 mRNA in cells. Namely, HIC‐5, NOX4, and mtROS formulate the molecular system that regulates MMP9 expression at the mRNA level. Notably, the axis operates specifically in cancer cells harboring the oncogenic mutations in H‐ or K‐*RAS*. Furthermore, a series of *in vivo* and *in vitro* experiments suggested that the system reduces invasiveness of cancer cells and mitigates their metastatic potential.

## Results

### HIC‐5 silencing promotes lung metastasis of MDA‐MB‐231 breast cancer cells *in vivo*

**Figure 1:**
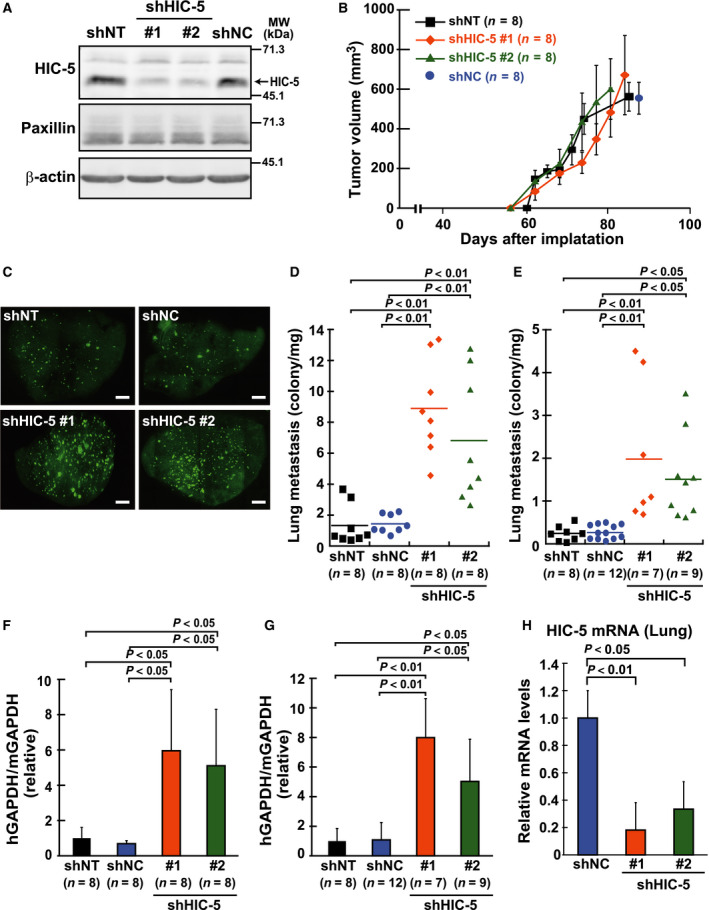
Hydrogen peroxide‐inducible clone‐5–silencing exacerbates lung metastasis of MDA‐MB‐231 breast cancer cells. Cells were established from the EGFP‐expressing MDA‐MB‐231 cells by lentiviral transduction of shRNA constructs (Materials and methods). The shRNAs incorporated in the constructs are two different nontargeting controls (shNT and shNC) and unrelated sequences specific for HIC‐5 (shHIC‐5 #1, #2; see Materials and methods). (A) Western blotting analysis of HIC‐5 and paxillin in cells. Total cell lysates were examined using the indicated antibodies. β‐actin was used as a loading control. (B–H) The shRNA‐expressing cells were inoculated into mammary fat pads of female NOD/SCID mice (B, C, D, F, and H) or injected intravenously in a tail vein of SCID mice (E, G). (B) Tumor volume in the mammary fat pads was monitored. Each data point represents the mean ± SD from eight xenografts. (C) Representative images of lung lobes excised from tumor‐bearing mice under florescence microscope. Images were taken at 20× magnification using a fluorescence microscope (BZ‐8100; Keyence, Osaka, Japan) and assembled into whole‐lobe images automatically using the image‐joint function of BZ‐analyzer (Keyence). GFP‐positive metastatic nodules are observed as dots. Scale bar, 200 μm. (D–G) Quantification of lung metastasis of cells by counting the number of nodules (D, E) and by qPCR (F, G), respectively. When the tumor volume reached approximately 1.0 cm^3^ in mammary fat pads (~ 80 days) (D) or 4 weeks after injection (E), the number of metastatic nodules visualized (C) was quantified in each lobe of the tumor‐bearing mice (Materials and methods). The total number of nodules from all lobes in a single mouse was plotted as a dot after being normalized against lung weight. The horizontal lines indicate the means from the indicated number of mice. (F, G) Total RNA was extracted from the lobe and human GAPDH mRNA was quantified by qPCR. The values were normalized against those of mouse GAPDH mRNA and shown as relative to the control lobe (shNT) (means ± SD). (H) mRNA levels of human HIC‐5 were examined in the same RNA sample with F by qPCR. The values were normalized with human GAPDH and shown as relative to the control (shNC) (mean ± SD, *n* = 8).

Hydrogen peroxide‐inducible clone‐5 is a multidomain protein that comprises four Leu‐ and Asp‐rich LD and LIM domains and serves as a molecular adaptor in a range of cellular activities from integrin signaling at focal adhesions to transcriptional activities in the nucleus 14, 15, 16. Our recent work suggested that HIC‐5 is involved in the regulation of anchorage dependence in cell growth 15, 17. In this study, we examined its involvement in tumor growth and/or metastasis using cells established from MDA‐MB‐231 breast cancer cells by viral transduction of shRNA for HIC‐5 (sequence #1 and #2). The cells expressing the shRNAs showed significantly reduced HIC‐5 expression levels compared to those of control cells similarly established with nontargeted shRNA sequences, NT or NC (Fig. 1A). The expression levels of paxillin, the protein most homologous to HIC‐5, remained unchanged in the HIC‐5–silenced cells. Tyrosine phosphorylation of paxillin at Y31 and activation‐dependent phosphorylation of SRC at Y416 were also unaffected by HIC‐5 knockdown (unpublished data). When tumorigenicity *in vivo* was observed by implanting these HIC‐5–silenced cells orthotopically into mammary fat pads of mice, HIC‐5–silenced cells formed tumors at rates comparable to those of the controls (Fig. 1B). The differences in tumor growth rates between cell lines were not statistically significant, suggesting that cancer cell growth at primary sites was virtually unaffected by HIC‐5 levels. However, lung metastasis from the sites was promoted by HIC‐5 knockdown (Fig. 1C, D and F). As shown in Fig. 1H, HIC‐5 knockdown was sustained in metastasized cells. A similar enhancement of lung metastasis was observed with cells injected from a tail vein (Fig. 1E and G). In both cases, we evaluated the metastasis by two methods, counting GFP‐positive nodules microscopically on lung surfaces (Fig. 1D and E) and quantifying human GAPDH mRNA, which represents cancer cells existing in the tissues of mice (Fig. 1F,G). These results suggest that HIC‐5 levels have a significant impact on the metastatic potential of cells.

### HIC‐5 silencing potentiates invasiveness of MDA‐MB‐231 breast cancer cells *in vitro*

**Figure 2:**
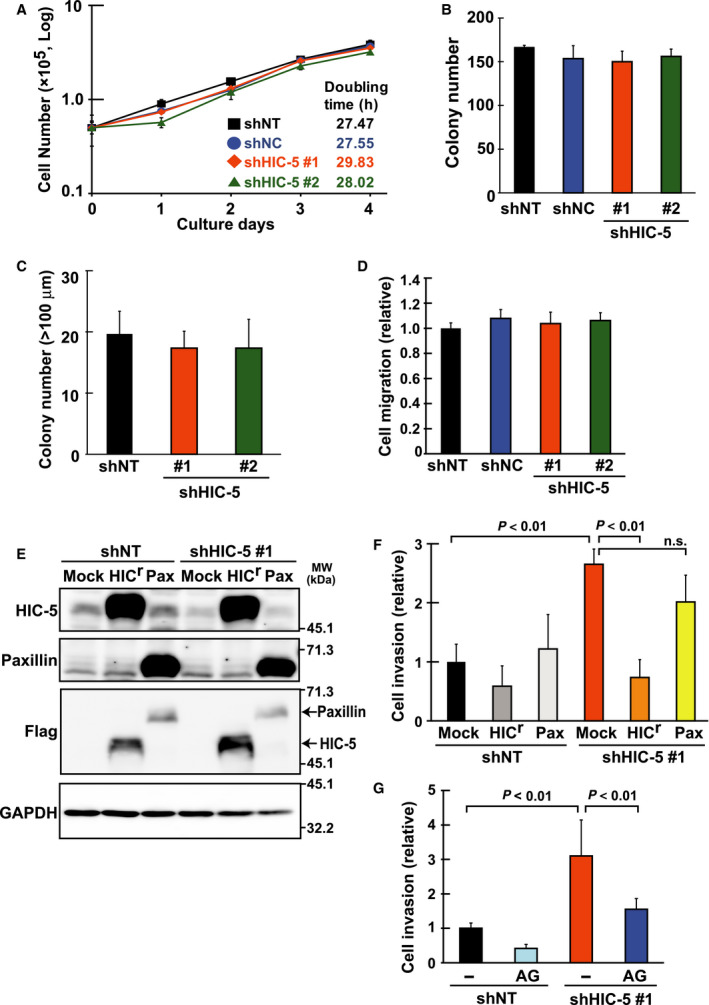
Hydrogen peroxide‐inducible clone‐5 silencing potentiates invasiveness but not growth properties of MDA‐MB‐231 breast cancer cells *in vitro*. The shRNA (shHIC‐5 #1, #2, or the control shNT, shNC)‐expressing cells were obtained from MDA‐MB‐231 cells by lentiviral transduction of the constructs (Materials and methods), and their growth properties (A–C), migratory (D), and invasive (F) abilities were examined. (A) Growth curve and doubling time of a population: 5 × 10^4^ cells were seeded in a well and the number of viable cells was counted each culture day. Doubling time was calculated from the slope of the graph. (B) Colony formation: cells were plated at a density of 500 cells/35‐mm‐diameter dish and the colony numbers were counted after 7 days. (C) Anchorage‐independent cell growth: cells were seeded at a density of 1 × 10^4^ cells per well in a polyHEMA‐coated six‐well plate with methylcellulose‐containing medium. After incubation for 3 weeks, whole‐well images were captured. The number of colonies (>100 μm) was counted using the imagej software. (D) Migration assay: cells were seeded at a density of 1 × 10^5^ cells per well in an upper chamber of tissue culture‐coated Transwell and after 20 h, those on the lower side of the membrane were stained with crystal violet and photographed under a light microscope. Cell migration was quantified as membrane area occupied by stained cells and shown as a ratio to the control (shNT). (E, F) The shRNA (shNT, shHIC‐5#1)‐expressing cells were infected with the lentiviral constitutive expression vectors (Mock, the empty vector; HIC ^r^, the Flag‐tagged shRNA‐resistant form of HIC‐5; Pax, a Flag‐tagged paxillin; see Materials and methods). After selection with antibiotics, the cells were analyzed by western blotting (E) and assayed for invasion (F). (E) Western blotting analysis of the Flag‐tagged HIC ^r^ and paxillin exogenously introduced in the cells with total cell lysates and the indicated antibodies. GAPDH was used as a loading control. (F) The cells were seeded and incubated using Matrigel‐coated Transwell chambers as in D. After removal of noninvading cells remaining on the upper side of the membrane, those on the lower surface were stained, and whole‐membrane images were captured. Cell invasion was evaluated as membrane area occupied by stained cells and shown as a ratio to the control (shNT/Mock). (G) The cells were seeded and incubated as in F in the presence or absence of an MMP inhibitor, AG3340 (2 μm). Cell invasion is shown as a ratio to the control (shNT/−). All values represent the means ± SD from three independent experiments measured in triplicate (n.s., not significant).

The metastatic potential of cells is sustained by various cellular activities. We subsequently examined the ability affected by HIC‐5 among those relevant to metastatic potential. A series of *in vitro* experiments suggested that HIC‐5 specifically influenced the invasiveness of cells (Fig. 2). Proliferative properties of cells, such as colony‐forming ability as well as anchorage‐independent growth, and migratory abilities, were not affected (Fig. 2A–D). However, the invasive ability of cells was evidently potentiated by HIC‐5 knockdown (Fig. 2F, shNT/Mock vs. shHIC‐5#1/Mock). The knockdown effect of shRNAs was neutralized by the coexpression of shRNA‐resistant HIC‐5 (HIC^r^) (Fig. 2E,F). Given that similar expression of paxillin instead of HIC^r^ only had a minor effect, it is unlikely that the neutralizing effect of HIC^r^ was a mere outcome of ectopic protein overexpression. Alternatively, it is likely to exclude the off‐target effect of shRNA and corroborate the involvement of HIC‐5 in the regulation of cell invasiveness. Collectively, these *in vitro* and *in vivo* observations consistently suggest that HIC‐5 regulates cell invasiveness, thereby modulating the metastatic potential of cancer cells. Notably, the increase in cell invasiveness under HIC‐5 knockdown was attenuated by the presence of a nontoxic dose of AG3340, an MMP inhibitor that potently blocks the activity of a subset of MMPs including MMP2 and MMP9 18 (Fig. 2G).

### MMP9 expression is upregulated by the stabilization of mRNA in HIC‐5–silenced cells

**Figure 3:**
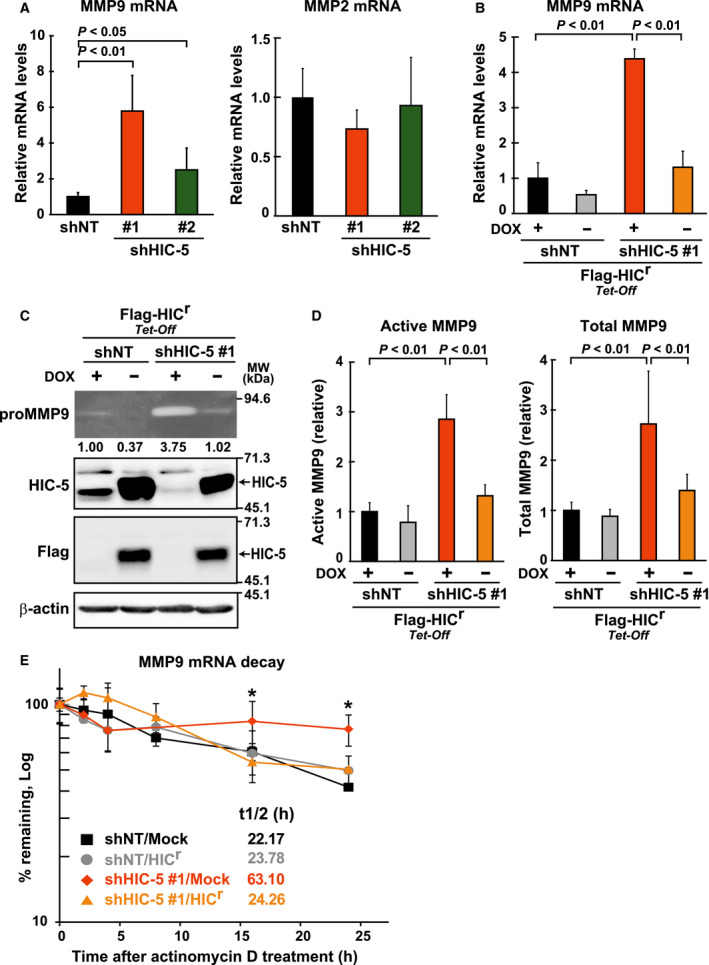
Endogenous HIC‐5 negatively regulates MMP9 mRNA expression. (A) MMP9 and MMP2 mRNA levels in the shRNA (shNT, shHIC‐5#1 and #2)‐expressing cells were quantified by qPCR. The values were normalized to the corresponding GAPDH in the same sample and are shown relative to the control (shNT) (means ± SD of triplicate samples from at least three independent experiments). (B–D) The cells expressing shHIC‐5 #1 or shNT were infected with the tetracycline‐regulated (Tet‐Off) lentiviral vector encoding the Flag‐tagged HIC ^r^ and selected with antibiotics (Materials and methods). The resistant cells were cultured for 24 h in the presence (DOX+) or absence (DOX−) of doxycycline (2 ng·mL^−1^). (B) MMP9 mRNA levels were examined by qPCR as in A. The values are relative to the control (shNT/DOX+) and plotted as in A (means ± SD of triplicate samples from at least three independent experiments). (C) The cells as in B were further cultured in serum‐free medium for another 24 h, and then conditioned medium and total cell lysate were subjected to gelatin zymography (the uppermost panel) and western blotting (lower panels), respectively. The gelatinolytic areas on zymography were quantified using the imagej software and normalized against the band intensity of β‐actin in the western blot of the corresponding sample. The ratio to the control (shNT/DOX+) is shown under the panel. (D) Activity of MMP9 in the conditioned medium obtained in C was quantified by QuickZyme Human MMP‐9 activity assay. The amount of active and total MMP9 is shown as the ratio to the control (shNT/DOX+) (means ± SD from two independent experiments measured in triplicate). (E) The shRNA (shNT, shHIC‐5#1)‐expressing cells introduced with the HIC ^r^‐ and Mock‐expressed vectors (constitutive) as in Fig. 2E were treated with actinomycin D (1 μg·mL^−1^) for the indicated time, and at each time point, the amount of MMP9 mRNA was determined by qPCR. After normalization to the amount of 18S rRNA, the percentages of remaining MMP9 mRNA were graphed (the value at 0 h defined as 100%) (means ± SD of triplicate samples from at least three independent experiments, *P < 0.01; vs. shNT/Mock). The half‐life (*t*_1/2_) of mRNA was calculated from the slope between the points of the graph and averaged.

The above observations led us to focus on MMP2 and MMP9, both of which are involved in cellular invasiveness and cancer metastasis 2, 4, 5. First, we observed that MMP9 mRNA, unlike MMP2 mRNA, was increased in HIC‐5–silenced cells, suggesting a role of HIC‐5 in the regulation of MMP9 expression (Fig. 3A). Similar observations were obtained under transient knockdown conditions with small interfering RNA (siRNA) for HIC‐5 (unpublished data). In parallel with the increase in mRNA, protein levels (proMM9 in Fig. 3C, and total in Fig. 3D) were elevated under these conditions (shNT/DOX [+] vs. shHIC‐5#1/DOX [+]), and were accompanied by an increase in the active form of MMP9 (Fig. 3D). These increases in MMP9 expression and activity were reversed by the coexpression of HIC^r^ (Fig. 3B–D; shHIC‐5#1/DOX [+] vs. [−]). Thus, HIC‐5 was shown to be involved in the regulation of MMP9 expression. Further investigation suggested that HIC‐5 influenced the stability of MMP9 mRNA; the half‐life of mRNA under actinomycin D treatment was extended by almost threefold in HIC‐5–silenced cells compared to the control (Fig. 3E, shNT/Mock vs. shHIC‐5#1/Mock), which was also reversed by coexpression of HIC^r^. The extension of the half‐life by HIC‐5 knockdown appeared to be specific to MMP9 mRNA, because that of c‐MYC mRNA in the same samples was similar to the control (unpublished data). In contrast to mRNA stability, the transcriptional activity of a −2.2‐kbp upstream element of the MMP9 gene was unchanged by HIC‐5 knockdown (unpublished data). Therefore, we concluded that HIC‐5 participated in the regulation of MMP9 expression by affecting mRNA stability.

### The stabilization of MMP‐9 mRNA is mediated by increased mtROS in HIC‐5–silenced cells

**Figure 4:**
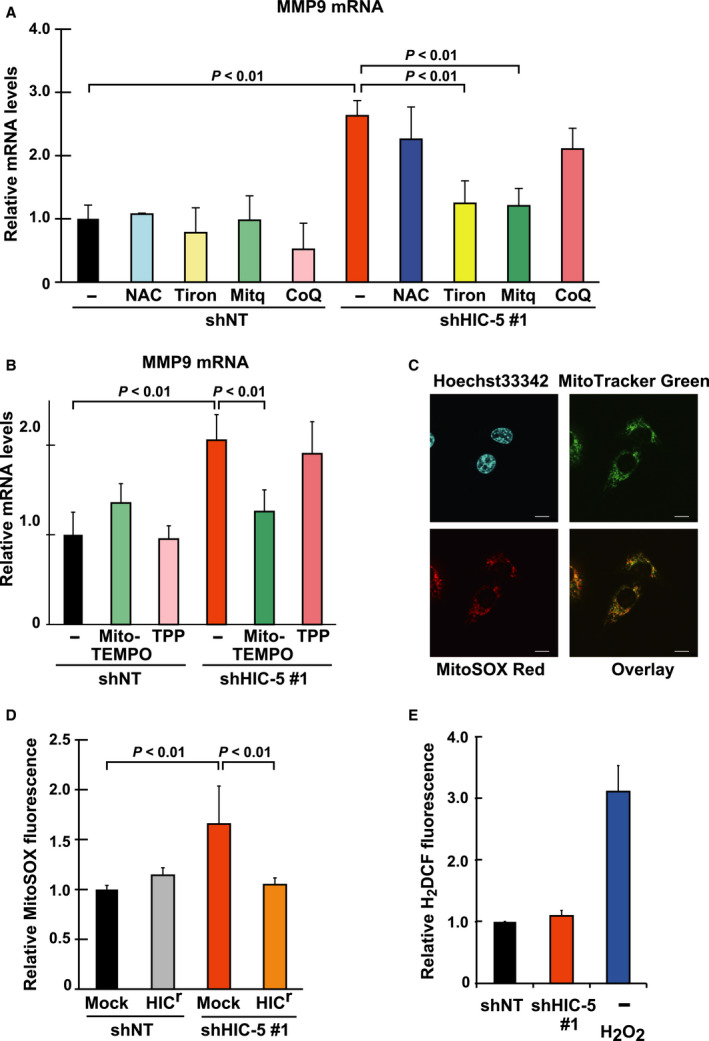
The upregulation of MMP9 mRNA is mediated by mtROS increased in HIC‐5–silencing cells. (A, B) The shRNA (shNT, shHIC‐5#1)‐expressing cells were treated with the following chemicals: A, NAC (2 mm), Tiron (1 mm), Mitq (0.5 μm), Mito‐TEMPO (10 μm), and CoQ (0.5 μm); and B, Mito‐TEMPO (10 μm) and TPP (10 μm)**.** After 24 h, MMP9 mRNA levels were quantified by qPCR. A ratio to the control (shNT/−) is shown (means ± SD of triplicate samples from at least three independent experiments)**.** (C) MDA‐MB‐231 cells were washed and incubated with 5 μm MitoSOX Red, 20 nm Mito Tracker Green FM, and 10 μm Hoechst 33342 for 10 min. After washing, laser scanning confocal microscopy was performed. Scale, 10 μm. (D, E) The shRNA (shNT, shHIC‐5#1)‐expressing cells introduced with HIC ^r^ and Mock as in Fig. 2E were analyzed for intracellular ROS levels with MitoSOX Red (D) and H_2_ DCFDA (E) by flow cytometry. The fluorescence intensities were normalized against that of calcein blue in individual cells. The values were obtained from at least 10 000 cells and the means ± SD are graphed as a ratio to the control [shNT/Mock (D) or shNT (E)]. Treatment with H_2_O_2_ (1 mm, 30 min) was used as positive control (E). Data are representative of two independent experiments with measurements in triplicate for each experiment.

Hydrogen peroxide‐inducible clone‐5 is a molecular adaptor that features redox‐sensitive expression and functions. In particular, it traffics in and out of the nucleus with the redox‐sensitive nuclear export signal and modulates cellular responses to redox changes 17, 19. In this study, we tested the possible association of the emerging role of HIC‐5 with redox changes in cells. With a set of redox‐modifying agents, we found that the increase in MMP9 mRNA under HIC‐5 knockdown conditions was sensitive to a subset of them (Fig. 4A). For example, mitoquinol (Mitq) and 1,2‐dihidroxybenzene‐3,5‐disulphonic acid (Tiron), which are mitochondria‐targeted and localized antioxidants, respectively 20, 21, 22, attenuated the increase in MMP9 mRNA by HIC‐5 knockdown. In contrast, coenzyme Q (CoQ), a nonmitochondria‐targeted quinone moiety used as a control of Mitq, and *N*‐acetyl cysteine (NAC), had only minor effects. These results suggested that the increase in MMP9 mRNA by HIC‐5 silencing was mediated by mtROS. To consolidate the evidence for the involvement of mtROS further, we used another mitochondria‐targeted antioxidant (2‐(2,2,6,6‐tetramethylpiperidin‐1‐oxyl‐4‐ylamino)‐2‐oxoethyl)triphenylphosphonium (Mito‐TEMPO) 23, and found that it similarly attenuated MMP9 induction by HIC‐5 knockdown; in contrast, the lipophilic cation, tripheylphosphonium (TPP), a moiety conjugated to deliver the antioxidant core, TEMPO, to the mitochondria, had no effects (Fig. 4B).

Consistent with these findings, ROS levels evaluated with the mitochondrial superoxide indicator MitoSOX, which was confirmed to be predominantly localized in the mitochondria in cells (Fig. 4C), were elevated under HIC‐5 knockdown conditions (Fig. 4D, shNT/Mock vs. shHIC‐5#1/Mock) and reversed by HIC^r^ coexpression. In contrast, ROS levels detected with H_2_DCF, an indicator of intracellular H_2_O_2_, were unchanged under HIC‐5 knockdown conditions (Fig. 4E). Therefore, it seems likely that HIC‐5 modulates mtROS levels (mainly superoxide), thereby affecting MMP9 mRNA stability.

### The increase in mtROS and consequent upregulation of MMP9 expression is mediated by the upregulation of NOX4 in HIC‐5–silenced cells

**Figure 5:**
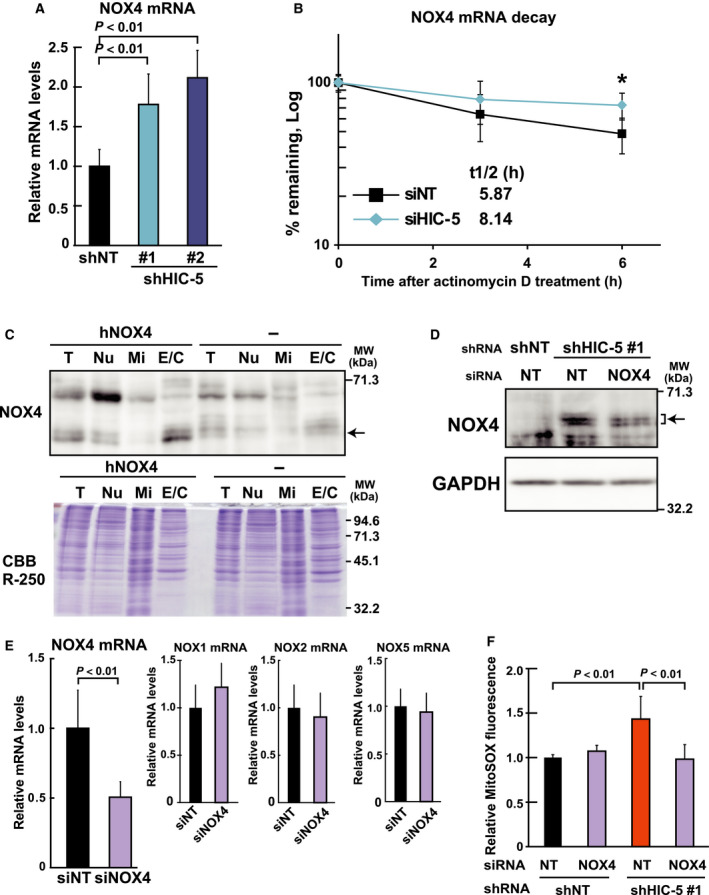
The increase in mtROS is mediated by the upregulation of NOX4 in HIC‐5–silenced cells. (A) Total RNA from the shRNA (shNT, shHIC‐5#1 and #2)‐expressing cells was analyzed for NOX4 mRNA levels by qPCR. A ratio to the control (shNT) is shown. (B) HMLER cells, which expressed NOX4 at high levels (see Fig. 7C), were transfected with siRNA for control (siNT) or HIC‐5 and 48 h later, treated with actinomycin D (1 μg·mL^−1^). The levels of NOX4 mRNA were determined by qPCR, and the remaining percentages at each time point were plotted (value at 0 h defined as 100%) as in Fig. 3E. *P < 0.01. (C) HEK293 cells with or without human NOX4 overexpression were subjected to fractionation and analyzed using western blotting as described in Materials and methods. T; total cell lysates, Nu; nuclei and unbroken cell containing, Mi; mitochondria rich, and E/C; microsomal and cytoplasmic fractions. The fractions containing equal amounts of protein were analyzed using western blotting with the antibody to NOX4. Proteins loaded and remaining in the gel were stained with Coomassie Brilliant Blue R‐250 (CBB R‐250). (D) The shRNA (shNT, shHIC‐5#1)‐expressing MDA‐MB‐231 cells were transfected with siRNA for control (NT) or NOX4. After 72 h, cells were disrupted and fractionated as in C. The E/C fractions were analyzed using western blotting. GAPDH was used as a loading control. (E) MDA‐MB‐231 cells were transfected with siRNA for control (NT) or NOX4, and 48 h later, NOX4, NOX1, NOX2, and NOX5 mRNA levels were determined by qPCR. Ratios relative to the controls (siNT) are shown. (F) The shRNA (shNT, shHIC‐5#1)‐expressing cells were transfected with siRNA for control (NT) or NOX4. After 48 h, ROS levels were determined with MitoSOX Red by flow cytometry as in Fig. 4D. The fluorescence intensities normalized against that of calcein blue in individual cells were obtained from at least 10 000 cells, and means ± SD are presented as a ratio to the control (shNT/NT). All data are representative of three independent experiments with measurements in triplicate for each experiment.

Subsequently, the mechanisms underlying the increase in mtROS in HIC‐5–silenced cells were analyzed. We focused on NADPH oxidases, major enzymes contributing to the production of ROS including $O_{2}^{-}$ and H_2_O_2_ in cells 24. Among the members, NOX4 is ubiquitously expressed in various types of cells and localized in cellular organelles including mitochondria, at least in part 25, 26. When NOX4 expression levels in the HIC‐5–silenced cells were compared with those in the control, we found that NOX4 expression was increased at the mRNA level by HIC‐5 knockdown (Fig. 5A). The levels of other members of the oxidase family, such as NOX1, NOX2, and NOX5, remained unaltered under the same conditions (unpublished data). The mRNA of NOX3 was undetectable in the cells. The increase in NOX4 expression may be due to mRNA stabilization; the half‐life of NOX4 mRNA was slightly but significantly (*P* = 0.0377) prolonged under knockdown conditions compared to the control (Fig. 5B). In contrast, the transcriptional activity of the −5.0‐kbp upstream element of the gene was scarcely increased, while the activity was upregulated by TGF‐β, an established transcriptional activator of NOX4 (unpublished data).

We next performed western blot analysis using a cytoplasmic fraction containing microsomes. To detect NOX4 protein with the antibody, it was necessary to eliminate some proteins that produced nonspecific bands with molecular weights close to that of NOX4 in MDA‐MB‐231 cells. As shown in Fig. 5C,D, cell fractionation showed that NOX4 proteins were mainly localized in the fraction (E/C; Fig. 5C), and the protein levels increased with HIC‐5 knockdown (Fig. 5D, shNT/NT vs. shHIC‐5#1/NT); this increase was attenuated in the presence of siRNA against NOX4. These results indicate that cellular NOX4 protein levels increased in response to increased mRNA upon HIC‐5 knockdown.

**Figure 6:**
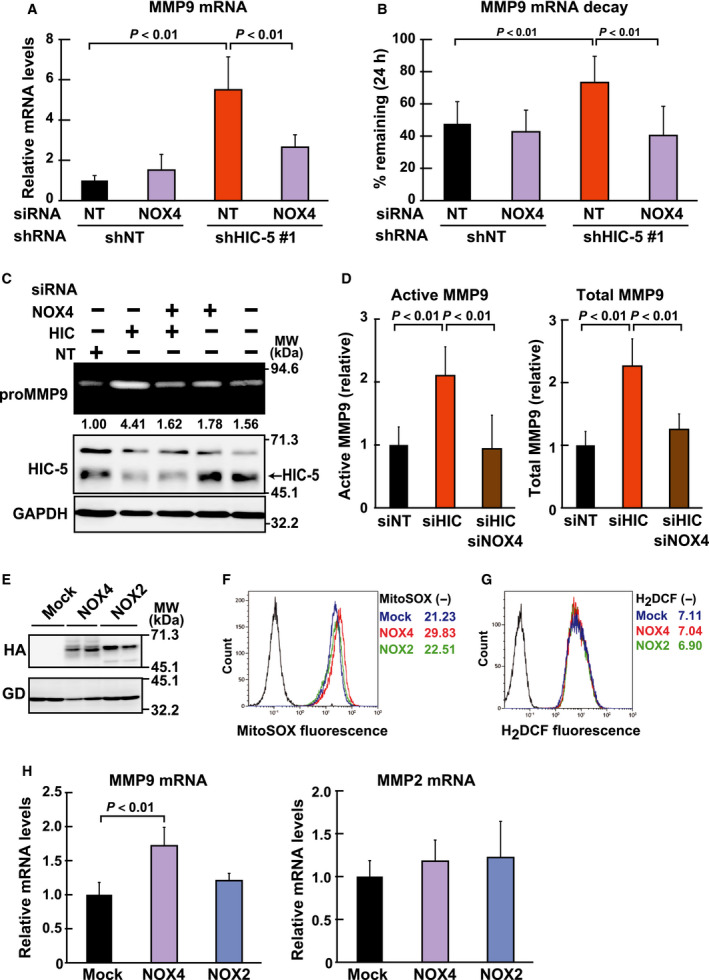
The upregulated NOX4 plays a role in the increase of MMP9 mRNA in HIC‐5–silenced cells. (A**,** B) The shRNA (shNT, shHIC‐5#1)‐expressing cells were transfected with the siRNAs (NT; control, NOX4), and incubated for 48 h. (A) The cells were analyzed for MMP9 mRNA levels by qPCR. A ratio to the control (shNT/NT) is shown (means ± SD of triplicate samples from at least three independent experiments). (B) The cells were further incubated with actinomycin D (1 μg·mL^−1^) for 24 h, and the amount of MMP9 mRNA was determined at 0 h and 24 h by qPCR. After normalization against the amount of 18S rRNA, ratios to the values at 0 h (remaining percentages at 24 h) were graphed (means ± SD of triplicate samples from at least three independent experiments). (C, D) The siRNAs (NT; control, HIC; HIC‐5, NOX4) were transfected into MDA‐MB‐231 cells in the combinations indicated. At 48 h post‐transfection, cells were transferred to serum‐free medium and cultured for another 24 h. In C, the conditioned medium and total cell lysates were prepared and analyzed by gelatin zymography (the uppermost panel) and western blotting (lower panels), respectively. Gelatinolytic areas were quantified using the imagej software and normalized against the band intensity of GAPDH in the western blot of the corresponding sample. A ratio to the control (NOX4−/HIC−/NT) is shown under the panel. In D, activity of MMP9 in the conditioned medium obtained in C was quantified by QuickZyme Human MMP‐9 activity assay. The amount of active and total MMP9 is shown as the ratio to the control (siNT). (E) The retroviral expression vectors for NOX4 and NOX2 (Materials and methods) were transfected into 293T cells, and protein expression was examined 48 h later by western blotting in duplicate with the antibodies as indicated. GAPDH (GD) was used as a loading control. (F‐H) MDA‐MB‐231 cells infected with the retroviral expression vectors for NOX4 and NOX2, or the empty vector (Mock) as in E were analyzed for ROS (F, G) and mRNA (H) levels. ROS levels were assessed with MitoSOX Red (F) and H_2_ DCFDA (G) by flow cytometry as in Fig. 4D and E. Representative histograms with the mean fluorescence intensities are shown. Data are representative of two independent experiments with measurements in triplicate for each experiment. MMP9 and MMP2 mRNA levels were quantified by qPCR (H). A ratio to the control (Mock) is shown (means ± SD of triplicate samples from at least three independent experiments). All data are representative of two independent experiments with measurements in triplicate for each experiment, unless otherwise noted.

To elucidate the role of the upregulated NOX4 under HIC‐5 knockdown, siRNA, which specifically knocked down NOX4 (Fig. 5E), was used to assess the effect of NOX4 depletion on the HIC‐5 knockdown‐mediated mtROS increase. As expected, MitoSOX‐monitoring ROS levels elevated by HIC‐5 knockdown returned to levels comparable to the control in the presence of the NOX4 siRNA (Fig. 5F). Accordingly, the increases in MMP9 expression and activity were also attenuated by NOX4 siRNA (Fig. 6A–D), which shortened the mRNA half‐life (Fig. 6B). These observations suggested that NOX4 mediated the effects of HIC‐5 knockdown on mtROS levels and MMP9 mRNA expression.

Consistent with the above observations, NOX4 exogenously expressed in the cells (Fig. 6E) faithfully recapitulated the effects of HIC‐5 knockdown. As shown in Fig. 6F–H, NOX4 expression resulted in increases in MitoSOX‐monitoring ROS (F) and MMP9 mRNA levels (H), whereas the levels of H_2_DCF‐monitoring ROS (G) and MMP2 mRNA (H) were barely changed. Unlike NOX4, expression of NOX2 showed no overt effect on either mtROS or MMP9 levels (Fig. 6F and H). Collectively, these loss‐ and gain‐of‐function experiments indicate that NOX4 is a downstream effector of HIC‐5.

### HIC‐5‐NOX4‐mtROS axis operates specifically in K‐ and H‐*RAS*‐mutated cancer cell lines

**Figure 7:**
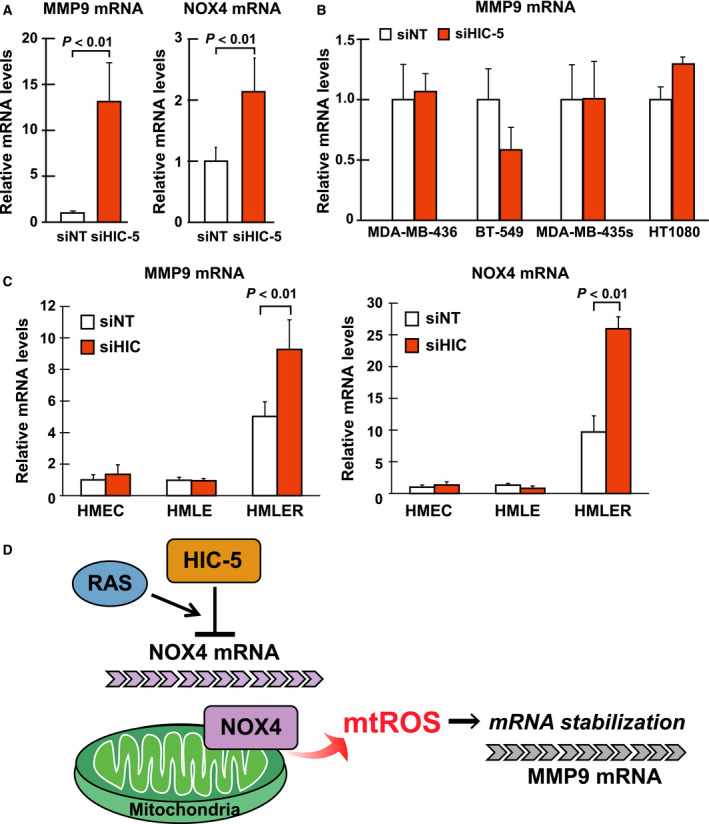
Hydrogen peroxide‐inducible clone‐5 function as a suppressor of NOX4 expression is dependent on oncogenic RAS signaling. (A) EJ‐1 cells were transfected with siRNA for control (siNT) or HIC‐5 (siHIC). After 48 h, total RNA from cells was analyzed for mRNA levels of MMP9 and NOX4 by qPCR. A ratio to the control (siNT) is shown. (B) The cell lines listed in Table 1 were transfected with siRNA and analyzed for mRNA levels of MMP9 as in A. (C) The siRNAs were transfected into the HMEC, HMLE, and HMLER cells (Materials and methods), and MMP9 and NOX4 mRNA levels were examined as in A. (D) HIC‐5‐NOX4‐mtROS axis: a new mode of regulation of MMP9 expression. The axis is engaged in the regulation of MMP9 mRNA stability. HIC‐5 suppresses NOX4 expression, which lowers MMP9 mRNA‐stabilizing mtROS levels. All values are means ± SD of triplicate samples from at least three independent experiments.

We then defined the attribute of cancer cells in which the HIC‐5‐NOX4‐mtROS axis regulates MMP9 expression. The HIC‐5–mediated upregulation of NOX4 and MMP9 mRNA was observed only in MDA‐MB‐231 (Figs. 3A and 5A) and EJ‐1 (Fig. 7A) among the six cancer cell lines studied (Table 1) (Fig. 7B). Both cell lines commonly bear mutations in H‐ and K‐*RAS*, respectively, suggesting that HIC‐5 function is coupled with activated H‐ or K‐RAS function. In contrast, in HT1080 cells harboring the N‐*RAS* mutation, the HIC‐5–mediated effect was not apparent. This may be due to cell origin differences (HT1080 is a fibrosarcoma of mesenchymal origin, whereas MDA‐MB‐231 and EJ‐1 are of epithelial origin). HIC‐5 is a multifaceted molecular adaptor that produces distinct scaffolds depending on localization and cell type 13. Alternatively, there might be a difference between N‐ and H‐, K‐*RAS* with respect to acting on HIC‐5.

**Table 1:** List of cancer cell lines

Origin	Cell line	Cell bank	Genetic mutation	Culture media
Breast cancer	MDA‐MB‐231	ATCC^®^ HTB‐26^™^	K‐RAS, BRAF, CDKN2A, TP53, NF2	DMEM, 10% FBS
	BT‐549	ATCC^®^ HTB‐122^™^	TP53, PTEN, RB1	RPMI, 10% FBS
	MDA‐MB‐436	ATCC^®^ HTB‐130^™^	RB1, BRCA1	DMEM, 10% FBS, 10 μg·mL^−1^ insulin
Bladder carcinoma	EJ‐1	JCRB: JCRB0710	H‐RAS, TP53	MEM, 10% FBS
Melanoma	MDA‐MB‐435s	ATCC^®^ HTB‐129^™^	TP53, BRAF, CDKN2A	DMEM, 10% FBS, 10 μg·mL^−1^ insulin
Fibrosarcoma	HT1080	JCRB: IFO50354	CDKN2A, N‐RAS, IDH1	MEM, 10% FBS

To clarify the linkage of the HIC‐5 function with activated H‐ and K‐*RAS* more directly, a human mammary epithelial cell line (hMEC) was transformed stepwise by transducing hTERT, SV40 early region and constitutively active H*‐RAS* sequentially, and obtained HMEC (hMEC + hTERT), HMLE (HMEC + SV40), and HMLER (HMLE + H*‐RAS*). Only after the introduction of H*‐RAS* did cell exhibit responsiveness to HIC‐5 knockdown and showed increased MMP9 and NOX4 mRNA (Fig. 7C, HMEC, HMLE vs. HMLER).

## Discussion

The present study describes the HIC‐5‐NOX4‐mtROS molecular axis, which formulates a new mode of regulation for MMP9 expression. The axis primarily regulates NOX4‐generating mtROS levels and targets MMP9 mRNA stability (Fig. 7D). As a result, the axis functions to decrease MMP9 expression at mRNA level. Importantly, the silencing of HIC‐5, the most upstream element and the determinant of the functionality of the axis, had a promoting effect on the metastatic potential of cancer cells (Figs 1 and 2). Accordingly, the functionality of the axis or HIC‐5 levels is expected to negatively correlate with the metastatic ability of tumors, particularly that of cancer cells harboring *RAS* mutations. In a clinical setting, HIC‐5 may serve as a prognostic factor for cancers with the RAS mutations (i.e., the lower HIC‐5 levels are linked to poorer prognoses).

In contrast to our conclusion highlighting HIC‐5 as a potential suppressor of metastasis (Fig. 1), a previous study suggested that HIC‐5 has a promoting role in cancer metastasis 27. In the study, MDA‐MB‐231 cells were treated with siRNA for HIC‐5 and intravenously injected from a tail vein. Under these conditions, lung metastasis was reduced by knockdown of HIC‐5. Regarding this discrepancy, it should be emphasized that we utilized shRNA instead of siRNA to ensure long‐term knockdown—HIC‐5 was knocked down sustainably in metastasized cells (Fig. 1H). Moreover, we assessed the metastasis of orthotopically inoculated cells as well as intravenously injected ones. In addition, we examined both the lung surface by fluorescence microscopy and the whole lung by qPCR (Fig. 1D–G). In contrast, in the previous study, the investigators focused on metastasis of the tail vein‐injected cells on the lung surface. On the basis of these differences, we argue that a long‐term reduction in HIC‐5 levels exacerbated metastasis and that HIC‐5 has a suppressive role in metastasis overall, especially at later stages. On the other hand, a transient silencing of HIC‐5 by siRNA could result in the inhibition of short‐term metastasis on the lung surface. HIC‐5 may possibly facilitate metastasis at initial stages by elevating RhoA signaling 27.

The functional core of the axis is the action of NOX4‐generating mtROS in the stabilization of MMP9 mRNA. This novel role of ROS has raised the possibility that the effects of ROS on cellular malignancy, such as migration, invasion, and epithelial–mesenchymal transition 28, 29, may be partly derived from their action upregulating MMP9. Our study also suggested that ROS exert their effects on MMP expression through regulation of mRNA stability in some cases. In the regulation of MMP, multiple processes have been described to be modified by ROS 10. Interestingly, NO was reported to destabilize MMP9 mRNA by inhibiting the expression of the mRNA‐stabilizing factor, HuR 30. Similarly, ROS may affect HuR expression, which is the subject of a future study.

The NOX4 contributes to MitoSOX‐detectable ROS production in cardiac myocytes and fibroblasts and in renal mesangial cells 31, 32, 33. NOX4 was similarly identified as the source of mtROS in this study. In these previous and our studies, NOX4 was suggested to be localized in the cytoplasmic fraction containing microsomes [endoplasmic reticulum (ER), this study] or to the mitochondria along with ER and plasma membrane in myocytes and other cells 25, 26, 34, suggesting multiple subcellular localizations of the NOX4 protein. In endothelial cells, NOX4 is detected in the ER 35, 36. Given that the ER and mitochondria are directly connected 37, any given protein could be detected in both compartments. Variations would depend on cell type and fractionation method. NOX4 might be a mitochondria‐associated membrane protein that mediates the functional cross‐talk between the two organelles 37.

Importantly, NOX4 has been associated with the malignant transformation of cells as well as MMP9 and mtROS. In particular, it was linked to the transformation of mammary epithelial cells 38. For example, it is overexpressed in the majority of breast cancer cell lines. Overexpression of NOX4 in already transformed breast tumor cells results in increased tumorigenicity 34. Notably, its contribution to malignant progression was reportedly through the generation of ROS in the mitochondria 34, which is consistent with evidence pertaining to its mitochondrial localization as above. Together with our results, it is possible that NOX4 contributes to the malignant progression of metastatic cells by increasing mtROS levels, thus acting to upregulate MMP9 expression.

The NOX4 is a constitutively active enzyme 39, whose activity is determined by mRNA levels 40, meaning that regulation of its expression at either transcriptional or post‐transcriptional levels is crucial for the prevention of ROS production by this enzyme under irrelevant circumstances. Currently, little is known about the regulation of NOX4 expression, especially at the post‐transcriptional level. Therefore, the emerging regulation of NOX4 mRNA expression by HIC‐5 in the axis may be an important control mechanism for this enzyme. A recent study reported a role of HIC‐5 in the post‐translational regulation of NOX4 through ubiquitination of the protein 41. These and our findings underline the importance of HIC‐5 as a regulator of NOX4 expression at the post‐transcriptional level. From a clinical point of view, these regulatory mechanisms for NOX4 expression are noteworthy in having the potential to provide opportunities for the development of therapeutic means that could decrease expression of MMP9, instead of inhibiting enzymatic activity *per se*. Inhibition of NOX4 expression hypothetically leads to reduced levels of mtROS, and ultimately the reduction in MMP9 expression, thereby serving as an alternative to MMP inhibitors.

## Materials and methods

### Cell lines, cell culture, reagents

Cancer cell lines were obtained from the American Type Culture Collection (Manassas, VA, USA) or Japanese Collection of Research Bioresources (Osaka, Japan) and maintained according to the instructions, with the exception that Leibovitz's L‐15 medium formulated for use in a CO_2_‐free atmosphere was replaced with Dulbecco’s modified Eagle’s medium (DMEM) in a 5% CO_2_ atmosphere as shown in Table 1. Human mammary epithelial cells (hMEC) were purchased from Lonza (Walkersville, MD, USA) and cultured as described previously 42. HEK293 cells was obtained from Human Science Research Resource Bank (Osaka, Japan) and maintained in MEM supplemented with 10% FBS and MEM nonessential amino acid (Life Technologies, Carlsbad, CA, USA).

BD Matrigel Matrix Growth Factor Reduced was obtained from BD Biosciences (Franklin Lakes, NJ, USA). Mitoquinol [10‐(6′‐ubiquinolyl) decyltriphenylphosphonium bromide] was synthesized as described previously 43. Ubiquinone‐10 (CoQ) was purchased from Wako Pure Chemical Industries Ltd. (Osaka, Japan). Other chemicals, including AG3340, 3(S)‐2,2‐dimethyl‐4‐[4‐pyridin‐4‐yloxy)‐benzenesulfonyl]‐thimorpholine‐3‐carboxylic acid hydroxyamide, were obtained from Sigma‐Aldrich (St Louis, MO, USA), unless otherwise noted.

### RNA interference

shRNA was expressed using the CS‐RfA‐FB or CS‐RfA‐EP lentiviral vector as described previously 42. The target sequences of nontargeting control shRNA [NT; CAA CAA GAT GAA GAG CAC CAA (SHC002)] and HIC‐5 #2 [TCT CTG ACT TCC GCG TTC AAA (TRCN0000020103)] were obtained from Mission shRNA (Sigma‐Aldrich) and that of negative control shRNA (NC; GGA ATC TCA TTC GAT GCA TAC) and HIC‐5 #1 (TCA GTT CAA CAT CAC AGA TGA) were from SABiosciences (a Qiagen company, Hilden, Germany).

siRNA (ON‐TARGETplusSMARTpool siRNA) was purchased from Thermo Fisher Scientific (Waltham, MA, USA). Transfection of siRNA (100 nm) was performed with X‐tremeGENE siRNA (Roche Applied Science, Penzberg, Upper Bavaria, Germany).

### Expression vectors

The cDNAs of human HIC‐5 and paxillin were amplified from pCG‐based constructs 44 and inserted into the CSII‐CMV‐MCS‐IRES2‐Bsd (constitutive) and CSII‐TREII vector (Tet‐Off) lentivirus vector 45 with a Flag tag sequence.

The HIC‐5 cDNA was converted to encode the shRNA‐resistant mutant by silent mutations introduced into the shHIC‐5 #1 target sequence (TCA GTT CAA CAT CAC AGA TGA) at the underlined positions using PrimeSTAR Mutagenesis Basal Kit (Takara Bio Inc., Kusatsu, Japan).

For expression vectors of HA‐tagged NOX4 and NOX2, the coding regions of the mouse genes were amplified from Mouse 17‐day Embryo Marathon^®^‐Ready cDNA (Takara Bio Inc.) and cloned into a retroviral pMXs‐IN vector 46.

cDNA encoding human NOX4 (hNOX4) was isolated from IMR‐90 by PCR and inserted into the lentiviral vector pCDH‐CMV‐MCS‐EF1‐GFP‐T2 A‐Puro (System Biosciences, PALA alto, CA, USA) to generate expression vector of hNOX4.

The constructs used for the transformation of hMEC were obtained as follows. The cDNA for constitutively active H*‐RAS* V12 (G12V) was generated by introducing point mutations into human H*‐RAS* cDNA amplified from the human mammary epithelial cell cDNA library using the PrimeSTAR Mutagenesis Basal Kit (Takara Bio Inc.) as above. The cDNA was expressed from a retroviral pMXs‐IP vector 47. SV40 large T antigen was expressed from pBABE‐zeo largeTgenomic kindly provided by B. Weinberg (Addgene plasmid # 1778) 48. All constructs were confirmed by sequencing.

### Cell establishment by viral transduction

The procedures for virus production and infection were essentially the same as that described previously 15. Successfully infected cells were selected, maintained in the medium supplemented with 10 μg·mL^−1^ blasticidin or 1 μg·mL^−1^ puromycin, and used for the experiments.

Human mammary epithelial cells derivatives (HMEC 42, HMLE, and HMLER) were generated by the sequential introduction of the catalytic subunit of human telomerase (hTERT), the SV40 early region, and H*‐RAS* V12 as described in previous literature 49. The established cells were cultured in MCDB 170 basal medium with supplements 42. Enhanced green fluorescent protein (EGFP)‐expressing MDA‐MB‐231 cells were previously described 43.

HEK293/hNOX4 was established by lentiviral transfer of the cDNA‐encoding hNOX4 as described previously 50.

### Western blots and subcellular fractionation

Western blotting has been described previously 43.

The following primary antibodies were used in this study: anti‐HIC‐5 antibody (61164) from BD Biosciences (San Jose, CA, USA); monoclonal antibodies to Flag (M2) (F‐1804), paxillin (P‐1093), and β‐actin (A‐1978) from Sigma‐Aldrich; and anti‐GAPDH antibody (MAB374) from Merck Millipore (Darmstadt, Germany). Anti‐HA antibody (12CA5) was obtained from Roche Applied Science (11583816001). Anti‐NADPH oxidase 4 antibody (UOTR1B493) was purchased from abcam (Cambridge, UK).

For subcellular fractionation, cells were disrupted by nitrogen cavitation (300 psi, 15 min) and separated into fractions by serial centrifugation as described elsewhere 46. The proteins in each fraction were solubilized and analyzed by western blotting.

### Cell proliferation, colony formation, and anchorage‐independent growth assays

To evaluate population doubling, cells were seeded at 5 × 10^4^ cells in six‐well plates, and the total cell number was counted with Countess^®^ (Thermo Fisher Scientific) each day after seeding. Cell viability was assessed by trypan blue staining.

For the colony formation assay, cells were plated at a density of 500 cells/35‐mm‐diameter dish. After 7 days, the developed colonies were stained with crystal violet and scored.

Anchorage‐independent cell growth was assayed as described previously 43 with slight modifications. In brief, a single‐cell suspension was prepared from monolayer culture by treatment with TrypLE™ Express (Thermo Fisher Scientific) and cultured at a density of 1.0 × 10^4^ cells per well in polyHEMA‐coated six‐well plates with growth medium supplemented with 1.0% methylcellulose. After 3 weeks, photographs were taken at 20 × magnification, and colonies with diameters larger than 100 μm were counted using the imagej software (Bethesda, MD, USA).

### Cell migration and invasion assays

Transwells (8‐μm pore size, Corning Inc., New York, USA) with tissue culture‐coated and Matrigel‐coated membranes were used for the migration and invasion assays, respectively. In both assays, cells were plated at a density of 1.0 × 10^5^ cells in an upper chamber of the Transwell. Culture medium in the upper chamber was serum free, and medium in the lower chamber was supplemented with serum as a chemoattractant. After 20 h of incubation, cells that did not migrate or invade through the pores were removed with a cotton swab, and those on the lower surface of the membrane were stained with 0.5% crystal violet. Cell migration and invasion were quantified by measuring the area occupied by stained cells on the membrane using the imagej software.

### Real‐time RT‐PCR (qPCR)

Total RNA was extracted from cultured cells and tissues, reverse‐transcribed into cDNA, and analyzed as previously described 42. The PCR primers are listed in Table 2.

**Table 2:** PCR primers

	Forward	Reverse
*Human*		
HIC‐5	5′‐CCACTCAGTTCAACATCACAGATG‐3′	5′‐TCAGACTGGTCCTCCTTCTGCT‐3′
MMP9	5′‐TTGACAGCGACAAGAAGTGG‐3′	5′‐CCCTCAGTGAAGCGGTACAT‐3′
MMP2	5′‐TGATGGCACCCATTTACACCT‐3′	5′‐AGAGCTCCCTGAATGCCCTTGA‐3′
NOX4	5′‐GCTGACGTTGCATGTTTCAG‐3′	5′‐CGGGAGGGTGGGTATCTAA‐3′
NOX1	5′‐TTGCAGCCGCACACTGA‐3′	5′‐GGCCACCAGCTTGTGGAA‐3′
NOX2	5′‐GTTCAGCTATGAGGTGGTGATGTT‐3′	5′‐TGGATGCGAAGGGTGTGA‐3′
NOX5	5′‐GCACCAGAAAAGAAAGCATACTTG‐3′	5′‐ATGTTGTCTTGGACACCTTCGA‐3′
GAPDH	5′‐CCAGGTGGTCTCCTCTGACTTC‐3′	5′‐GTGGTCGTTGAGGGCAATG‐3′
18S rRNA	5′‐CATGCATGTCTAAGTACGCACG‐3′	5′‐GCGACCAAAGGAACCATAACTG‐3′
*Mouse*		
GAPDH	5′‐ATGTGTCCGTCGTGGATCTGA‐3′	5′‐ATGCCTGCTTCACCACCTTCT‐3′

### Enzymatic assay of MMP9

Zymography was performed as described previously 51 with minor modifications. Cells were incubated in serum‐free medium containing 0.1% BSA, and the conditioned medium was collected after 24 h. Following centrifugation (3000 ***g***, 5 min at 4 °C) to remove cells and debris, the medium was separated by electrophoresis on 7% polyacrylamide gels containing 1 mg·mL^−1^ gelatin as a substrate after normalization to the total protein from the cells left in the same well. After Coomassie blue staining, the gelatinolytic areas were quantified using the imagej software on the acquired images.

Activity of MMP9 in the conditioned medium collected as above was quantified by QuickZyme Human MMP‐9 activity assay (QucikZyme BioSciences, Leiden, the Netherlands) according to the manufacturer's instructions. In brief, the activity of MMP9 (active MMP9) was measured with a prodetection enzyme. In parallel, the total amount of MMP9 (total MMP9), which was evaluated as the activity after the treatment with 4‐aminophenylmercuric acetate, an activator of pro‐MMP, was determined.

### ROS measurement and confocal microscopy

Cells were incubated with 5 μm MitoSOX Red (Thermo Fisher Scientific) and 1 μm calcein blue (Thermo Fisher Scientific) for 30 min at 37 °C after withdrawal of cells from the culture dish with TrypLE™ Express. After washing, cells were stained with 1 μg·mL^−1^ propidium iodide (Sigma‐Aldrich). Data from at least 10 000 events were acquired using the MoFlo Astrios EQ (Beckman Coulter, Inc., Fullerton, CA, USA) and analyzed by the kaluza analysis software 1.3 (Beckman Coulter). The excitation (ex) and emission (em) wavelengths were as follows: MitoSOX Red (ex 488 nm, em 576/21 nm), calcein blue (ex 405 nm, em 448/59 nm), and propidium iodide (PI) (ex 488 nm, em 644/22 nm). Debris and dead cells were excluded by forward scatter, side scatter, and PI gating. Color compensation was applied to properly analyze multicolor data. The level of mitochondria‐associated ROS was evaluated as the fluorescence intensity of MitoSOX Red normalized to that of calcein blue in individual cells.

The quantification of intracellular ROS levels using H_2_DCFDA (Thermo Fisher Scientific) was performed as described previously 43.

Subcellular localization of MitoSOX was examined by incubating cells with 5 μm MitoSOX Red, 20 nm Mito Tracker Green FM, and 10 μm Hoechst 33342 at 37 °C in the dark for 10 min. After washing, laser scanning confocal microscopy was performed using FV10i‐LIV confocal microscope (Olympus Corporation, Tokyo, Japan).

### Animal studies

All animal experiments were performed according to a protocol approved by the Institutional Animal Care and Use Committee at Showa University. Five‐week‐old female C.B‐17/Icr‐scid/scidJcl (SCID) and NOD.CB17‐*Prkdc* ^*scid*^/J (NOD/SCID) mice were purchased from CLEA Japan, Inc. (Tokyo, Japan) and Charles River Laboratories International, Inc. (Wilmington, MA, USA), respectively.

The procedures for orthotopic injection and monitoring tumor growth using NOD/SCID mice have been described previously 43. For tail vein injection, 1 million cells in 100 μL of Hank's balanced salt solution were injected into the lateral tail vein of SCID mice.

To quantify pulmonary metastasis, tumor‐bearing mice were euthanized and necropsied when the tumors reached approximately 1.0 cm^3^ in volume. In the case of tail vein injection, 4 weeks after injection, all mice were euthanized and necropsied. Whole‐lobe images were taken at 20× magnification using a fluorescence microscope (BZ‐8100; Keyence, Osaka, Japan), and the fluorescent metastatic nodules in the images were all counted using the imagej software, and normalized against the corresponding lung weight. After photography, the lobes were snap frozen in liquid nitrogen and stored at −80 °C. Total RNA was extracted from the frozen right lower lobe using QuickGene RNA tissue kit S II (KuRaBo, Osaka, Japan) according to manufacturer's recommendations.

### Statistical analysis

All results were expressed as the mean ± SD, unless otherwise noted. Differences between samples and paired controls were analyzed using the two‐tailed Student's *t*‐test. *P* < 0.05 was considered statistically significant. In multiple comparisons, data were analyzed using one‐way ANOVA with *post hoc* Dunnett or Bonferroni tests to identify datasets that differed from control data.

## Conflict of interest

The authors have no conflicts of interest with the contents of this article.

## Author contributions

KM designed, performed, and analyzed the experiments in all figures and wrote the paper. TU performed and analyzed the *in vivo* experiments. TY prepared the constructions and provided the assistance to the *in vivo* experiments. YM constructed the virus vectors and provided the assistance to the analysis of the cell phenotypes *in vitro*. FI designed and constructed virus vectors and contributed to the preparation of the paper. MK constructed luciferase reporters and contributed to the preparation of the paper. MS conceived and coordinated the study and wrote the paper. All authors reviewed the results and approved the final version of the manuscript.
